# Association of the Triglyceride–Glucose Index with Premature Myocardial Infarction in a Propensity Score-Matched Non-Diabetic Population

**DOI:** 10.3390/diagnostics16182999

**Published:** 2026-09-16

**Authors:** Cagatay Onal, Burak Ayca

**Affiliations:** 1 Department of Cardiology, Private Gazi Hospital, Nevzat Guzelirmak Street No. 29 Alsancak/Kahramanlar, Izmir 35230, Turkey; 2 Department of Cardiology, Buca Seyfi Demirsoy Training and Research Hospital, Izmir 35148, Turkey

**Keywords:** triglyceride–glucose index, premature myocardial infarction, insulin resistance, non-diabetic population, young adults, cardiovascular risk

## Abstract

**Background/Objectives**: Evidence regarding the triglyceride–glucose (TyG) index and premature myocardial infarction (MI) in non-diabetic adults remains limited. We evaluated whether pre-event TyG values were associated with premature MI in a propensity score-matched population. **Methods**: We retrospectively compared premature MI cases with non-diabetic controls undergoing coronary computed tomography angiography (CCTA) showing either no plaque or mild/non-obstructive plaque. Propensity-based 1:1 matching on age, sex, body mass index (BMI), smoking, and hypertension resulted in 114 matched pairs. Conditional logistic regression, dose–response analyses, restricted cubic spline (RCS) modeling, and incremental discrimination analyses were performed. **Results**: After propensity score matching, 228 patients (114 premature MI cases and 114 controls) were analyzed. The TyG index was independently associated with premature MI in conditional logistic regression (adjusted OR 2.704, 95% CI 1.408–5.192; *p* = 0.003). Compared with the lowest TyG tertile, the odds of premature MI increased progressively across higher tertiles (T2 vs. T1: adjusted OR 2.859, 95% CI 1.376–5.940; *p* = 0.005; T3 vs. T1: adjusted OR 4.424, 95% CI 1.859–10.531; *p* < 0.001). Restricted cubic spline analysis demonstrated a significant overall association between the TyG index and premature MI (*p* = 0.003), with no evidence of nonlinearity (*p* = 0.286). Adding the TyG index to the clinical model increased the AUC from 0.647 to 0.696 (ΔAUC = 0.050; DeLong *p* = 0.046). Matched-pair bootstrap analysis also supported an improvement in discrimination (95% CI for ΔAUC 0.002–0.096; *p* = 0.034). **Conclusions**: Higher pre-event TyG values were independently associated with premature MI in non-diabetic adults, with a graded association across TyG levels and no significant evidence of nonlinearity. Incremental discrimination beyond the clinical model was modest but statistically supported by both DeLong and matched-pair bootstrap analyses. Prospective multicenter validation is warranted.

## 1. Introduction

Premature myocardial infarction (MI) represents an increasingly important clinical and public health challenge despite substantial advances in cardiovascular prevention and treatment strategies. Recent evidence suggests that the incidence of premature coronary artery disease (CAD) and acute MI among young adults has increased over recent decades, particularly in individuals with multiple modifiable cardiovascular risk factors [1,2]. Although younger patients generally exhibit less extensive coronary atherosclerosis compared with older populations, premature CAD remains associated with substantial long-term morbidity, recurrent ischemic events, and premature mortality [3]. Smoking, excess body weight, lipid abnormalities, and a positive family history of CAD are frequently encountered in individuals presenting with premature MI, suggesting a substantial role of adverse cardiometabolic profiles in the development of premature CAD [4].

Cardiometabolic disturbances, particularly obesity and dysregulated glucose homeostasis, have been linked to adverse cardiovascular outcomes and poorer long-term prognosis in patients with premature MI [5]. Insulin resistance contributes to endothelial dysfunction, inflammation, oxidative stress, dyslipidemia, and accelerated atherosclerosis, thereby promoting cardiovascular diseases [6]. Moreover, insulin resistance has been linked to greater coronary lesion complexity and poorer clinical outcomes in patients presenting with acute coronary syndromes (ACS) [7].

The triglyceride–glucose (TyG) index, calculated from fasting triglyceride and glucose concentrations, has been proposed as a practical marker reflecting insulin resistance [8]. Growing evidence suggests that higher TyG values are linked to the extent of atherosclerotic disease and a higher prevalence of obstructive CAD [9,10]. In addition, increased TyG index levels have been related to adverse cardiovascular outcomes among patients with premature CAD and ACS [11,12]. However, evidence addressing the relationship between the TyG index and premature MI in non-diabetic young adults is limited. To address this gap, we evaluated whether the TyG index is associated with premature MI in a propensity score-matched (PSM) cohort of non-diabetic individuals.

## 2. Materials and Methods

### 2.1. Study Design and Population

In this retrospective matched case-control analysis, we evaluated data between January 2018 and December 2024. Patients with acute premature MI were consecutively identified and assessed for eligibility using prespecified criteria. The control group comprised individuals referred for coronary computed tomography angiography (CCTA) during the same period because of atypical chest pain, preoperative cardiovascular assessment, a family history of premature CAD, or cardiovascular risk evaluation. Control subjects could have either no coronary plaque or mild/non-obstructive plaque on CCTA. Coronary stenosis severity was classified according to the CAD-RADS framework, and controls were restricted to CAD-RADS 0–2 (<50% luminal stenosis) [13]. The presence of coronary plaque alone was not considered an exclusion criterion. Controls with mild or non-obstructive coronary plaque were retained to avoid selecting an artificially low-risk, entirely plaque-free comparison group and to better reflect the clinically referred source population.

To minimize the effects of overt diabetes and glucose-lowering treatment on the TyG index, individuals with diabetes mellitus were excluded from both groups. Diabetes mellitus was defined by a documented history of diabetes and/or ongoing glucose-lowering therapy; individuals meeting either criterion were excluded from both groups. Additional exclusions included prior MI or coronary revascularization, active infection or chronic inflammatory disease, malignancy, severe hepatic or end-stage renal disease, unavailable fasting glucose or triglyceride data, and use of fibrates or gemfibrozil.

**Figure 1:**
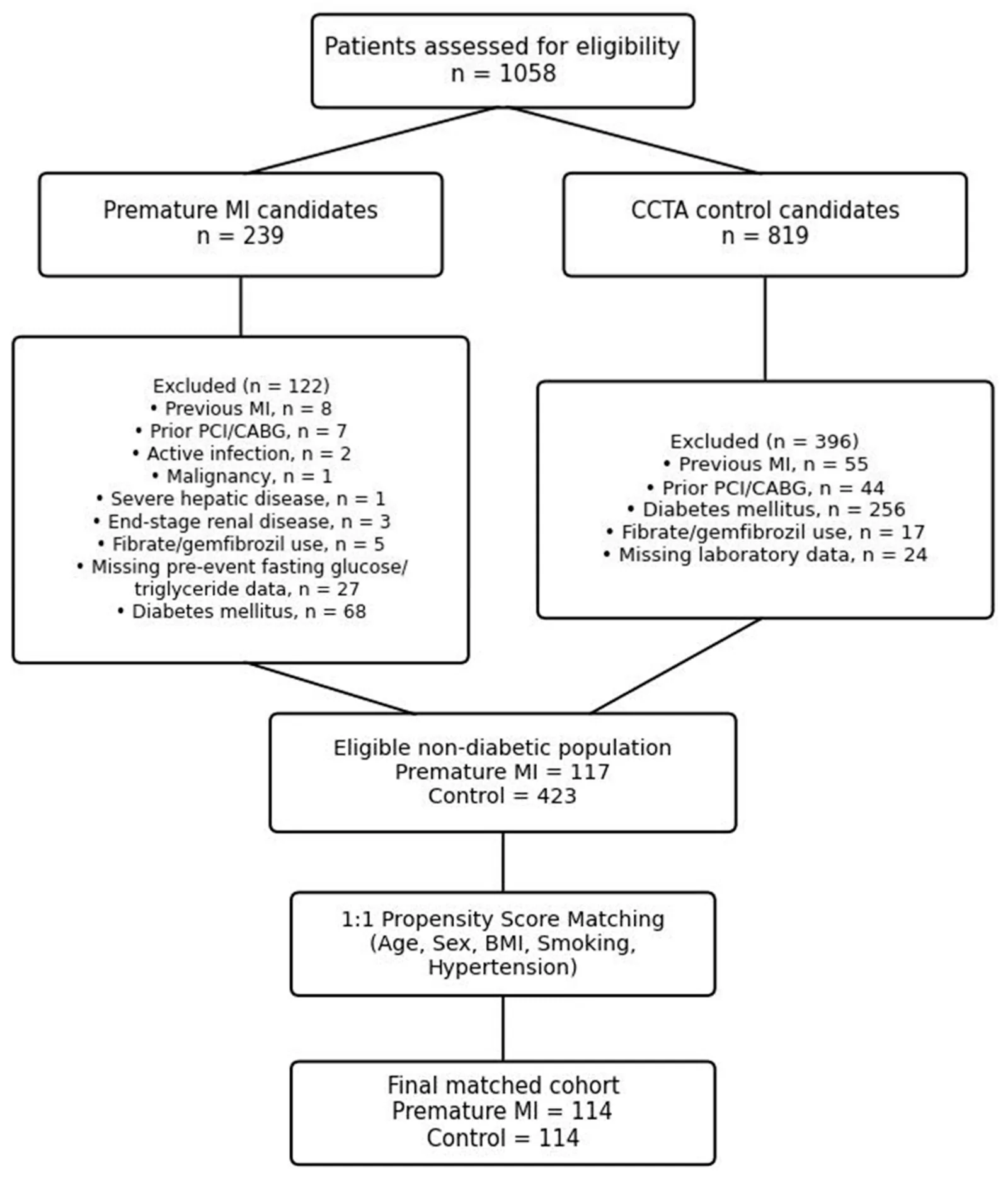
Study flowchart illustrating patient selection, exclusion criteria, and propensity score matching.

Overall, 1058 individuals were assessed for eligibility, comprising 239 premature MI candidates and 819 CCTA control candidates. After screening, 117 premature MI cases and 423 controls underwent 1:1 nearest-neighbor PSM based on age, sex, body mass index (BMI), smoking, and hypertension, using exact sex matching and a 0.20-SD caliper of the propensity-score logit. The final matched cohort included 114 patients with premature MI and 114 control subjects. The study selection and matching process is summarized in Figure 1.

### 2.2. Definitions

Premature MI was defined as MI occurring at ≤45 years in men and ≤55 years in women [14,15]. Acute MI was identified according to the Fourth Universal Definition of Myocardial Infarction [16], based on dynamic cardiac troponin changes together with evidence of myocardial ischemia. Cases were categorized as ST-segment elevation myocardial infarction (STEMI) or non-ST-segment elevation myocardial infarction (NSTEMI) in accordance with current European Society of Cardiology criteria [17].

### 2.3. Data Collection

Data on patient characteristics, cardiovascular risk factors, medications, laboratory measurements, and angiographic findings were extracted from the institutional electronic records. The main analyses included age, sex, BMI, smoking, hypertension, statin use, fasting glucose, lipid profile parameters, serum creatinine, hemoglobin, complete blood count parameters, and C-reactive protein (CRP).

In the premature MI group, TyG was derived from the most recent fasting glucose and triglyceride values available within 6 months before the index MI; patients lacking such pre-event measurements were excluded. For control subjects, fasting glucose and triglyceride measurements obtained during the outpatient evaluation within one week preceding CCTA were used. In the control group, CCTA findings were reviewed to confirm the absence of obstructive CAD; individuals with either no coronary plaque or only mild/non-obstructive plaque were eligible for inclusion. For the premature MI group, MI phenotype (STEMI or NSTEMI), culprit artery, angiographic extent of coronary disease, and revascularization strategy were additionally recorded.

### 2.4. TyG Index Calculation

The TyG index was calculated from fasting triglyceride and fasting plasma glucose concentrations using the following formula: TyG index = ln [fasting triglyceride (mg/dL) × fasting glucose (mg/dL)/2].

### 2.5. Statistical Analysis

Continuous data are reported as mean ± standard deviation or median (interquartile range), and categorical data as number and percentage. Normality was assessed with the Shapiro–Wilk test. In the matched cohort, continuous variables were compared using paired *t*-tests or Wilcoxon signed-rank tests, and categorical variables with McNemar’s test. Within the premature MI group, TyG comparisons were performed using the Mann–Whitney U or Kruskal–Wallis test, as appropriate.

PSM was conducted 1:1 by nearest neighbor search without replacement using age, sex, BMI, smoking, and hypertension, with exact matching on sex and a 0.20-SD caliper of the propensity-score logit. Balance was assessed by standardized mean differences, with |SMD| < 0.10 indicating adequate balance for matched covariates.

Associations between TyG and premature MI were evaluated by univariable and multivariable conditional logistic regression, with matched-pair ID used as the stratum. Multivariable models were adjusted for age, BMI, smoking status, HDL-C, and serum creatinine. TyG was modeled continuously and by tertiles derived from its distribution in the entire matched cohort (T1 < 8.84, T2 8.84–9.24, and T3 > 9.24). Additional analyses examined TyG per 1-SD increase, linear trend across tertiles, and restricted cubic spline modeling accounting for the matched design, with three knots at the 10th, 50th, and 90th percentiles. The median TyG value served as the reference, and the spline model was adjusted for age, BMI, smoking status, HDL-C, and serum creatinine.

Discrimination was assessed by ROC analysis. A clinical model comprising age, BMI, smoking status, HDL-C, and serum creatinine was compared with the same model including TyG. AUCs were compared using DeLong’s test, with matched-pair bootstrap resampling (2000 iterations) as a sensitivity analysis. Two-sided *p* values < 0.05 were considered significant. Analyses were performed using IBM SPSS Statistics version 23.0 and R software version 4.6.0.

## 3. Results

### 3.1. Study Population and Baseline Characteristics

A total of 1058 individuals were screened for eligibility (239 premature MI candidates and 819 CCTA control candidates). After the predefined exclusion criteria were applied, 117 patients with premature MI and 423 control subjects remained eligible for matching. One-to-one PSM yielded 114 matched pairs, resulting in a final analytical cohort of 228 participants (Figure 1). Balance for all variables included in the propensity score model was adequate after matching, with absolute SMDs below 0.10 (Supplementary Table S1). Among patients with premature MI, the median interval between the pre-event laboratory measurement and the index MI was 97 days (IQR, 65–128 days).

Baseline characteristics are summarized in Table 1. Age, BMI, sex, smoking, hypertension, and statin use were comparable between groups. Total cholesterol, LDL-C, hemoglobin, platelet count, lymphocyte count, and CRP also did not differ significantly. In contrast, patients with premature MI had higher fasting glucose, triglyceride, serum creatinine, neutrophil, and monocyte values and lower HDL-C levels than matched controls. TyG values were greater in the premature MI group than in matched controls [9.18 (IQR 8.88–9.40) vs. 8.93 (IQR 8.54–9.24), *p* < 0.001] (Table 1).

### 3.2. Clinical Phenotype and Angiographic Characteristics

Clinical presentation and angiographic findings in the premature MI group are shown in Table 2A. Among the 114 patients, 57 (50.0%) presented with STEMI and 57 (50.0%) with NSTEMI. The left anterior descending artery was the most frequent culprit vessel [56 patients (49.1%)], followed by the right coronary artery [28 (24.6%)] and left circumflex artery [25 (21.9%)]; another culprit location was recorded in 5 patients (4.4%). Angiographic disease extent was classifiable in 111 patients, of whom 97 (87.4%) had single-vessel disease, and 14 (12.6%) had multivessel disease. PCI was the predominant treatment strategy [110 patients (96.5%)], whereas 1 patient (0.9%) underwent CABG and 3 (2.6%) were treated medically.

The association between the TyG index and MI phenotype and angiographic characteristics is shown in Table 2B. TyG values did not differ significantly between STEMI and NSTEMI [9.09 (IQR 8.85–9.37) vs. 9.23 (IQR 8.91–9.46), *p* = 0.347], between single-vessel and multivessel disease [9.18 (IQR 8.87–9.40) vs. 9.24 (IQR 9.00–9.54), *p* = 0.401], or across culprit-vessel categories (*p* = 0.138).

### 3.3. Univariable and Multivariable Regression Analyses

Univariable conditional logistic regression results are presented in Table 3. Higher fasting glucose, serum creatinine, and monocyte count were significantly associated with premature MI. The TyG index was also significantly associated with premature MI (OR 2.731, 95% CI 1.526–4.886; *p* < 0.001). Male sex was not estimable because there were no sex-discordant matched pairs.

In multivariable conditional logistic regression, the TyG index remained independently associated with premature MI after adjustment for age, BMI, smoking status, HDL-C, and serum creatinine. In Model A, each one-unit increase in the TyG index was associated with higher odds of premature MI (adjusted OR 2.704, 95% CI 1.408–5.192; *p* = 0.003). In Model B, compared with the lowest TyG tertile (T1), both T2 (adjusted OR 2.859, 95% CI 1.376–5.940; *p* = 0.005) and T3 (adjusted OR 4.424, 95% CI 1.859–10.531; *p* < 0.001) were associated with progressively higher odds of premature MI (Table 4).

### 3.4. Additional Dose–Response Analyses

Additional dose–response analyses are summarized in Table 5. Each 1-SD increase in the TyG index was associated with a 69.6% increase in the adjusted odds of premature MI (adjusted OR 1.696, 95% CI 1.199–2.398; *p* = 0.003). A significant graded association was also observed across TyG tertiles. Compared with T1, the adjusted odds of premature MI were higher in T2 (OR 2.859, 95% CI 1.376–5.940; *p* = 0.005) and T3 (OR 4.424, 95% CI 1.859–10.531; *p* < 0.001). When TyG tertiles were modeled as an ordinal variable, each one-tertile increase was associated with approximately twofold higher odds of premature MI (adjusted OR 2.159, 95% CI 1.403–3.321; *p* for trend <0.001).

### 3.5. Incremental Discrimination Analysis

**Figure 2:**
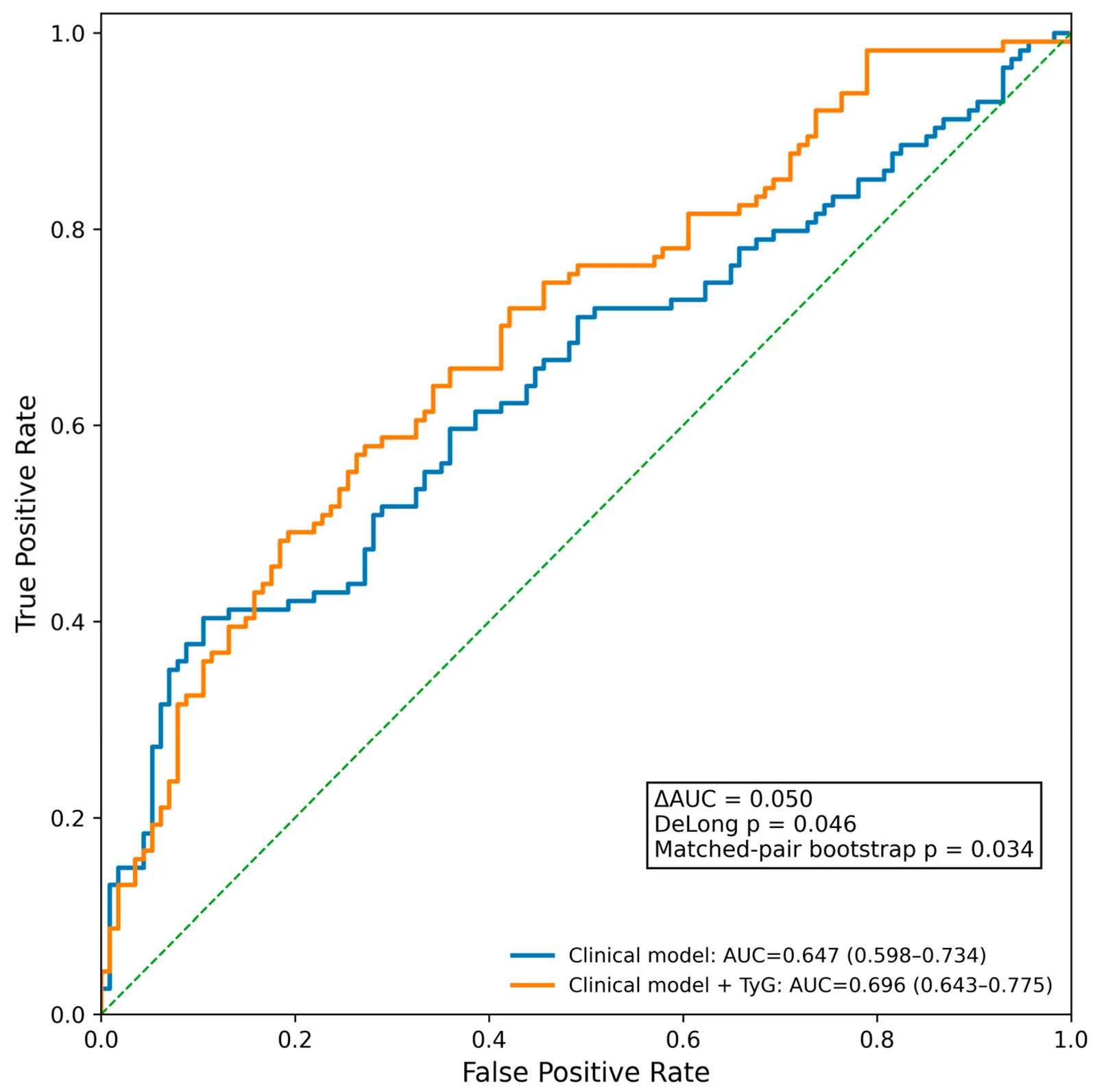
Receiver operating characteristic curves for discrimination of premature myocardial infarction. The clinical model included age, body mass index, smoking status, HDL-C, and serum creatinine. The addition of the TyG index increased the AUC from 0.647 to 0.696 (ΔAUC = 0.050; DeLong *p* = 0.046; matched-pair bootstrap *p* = 0.034). AUC, area under the receiver operating characteristic curve; HDL-C, high-density lipoprotein cholesterol; TyG, triglyceride–glucose. The dashed green line represents the line of no discrimination (AUC = 0.5).

The clinical model, including age, BMI, smoking status, HDL-C, and serum creatinine, yielded an AUC of 0.647 (95% CI, 0.598–0.734). After the addition of the TyG index, the AUC increased to 0.696 (95% CI, 0.643–0.775), corresponding to a ΔAUC of 0.050. The difference was statistically significant according to the DeLong test (*p* = 0.046). Matched-pair bootstrap analysis with 2000 resamples yielded a 95% CI for the ΔAUC of 0.002–0.096 (*p* = 0.034), also supporting a modest incremental improvement in discrimination after the addition of the TyG index (Table 6; Figure 2).

### 3.6. Restricted Cubic Spline Analysis

**Figure 3:**
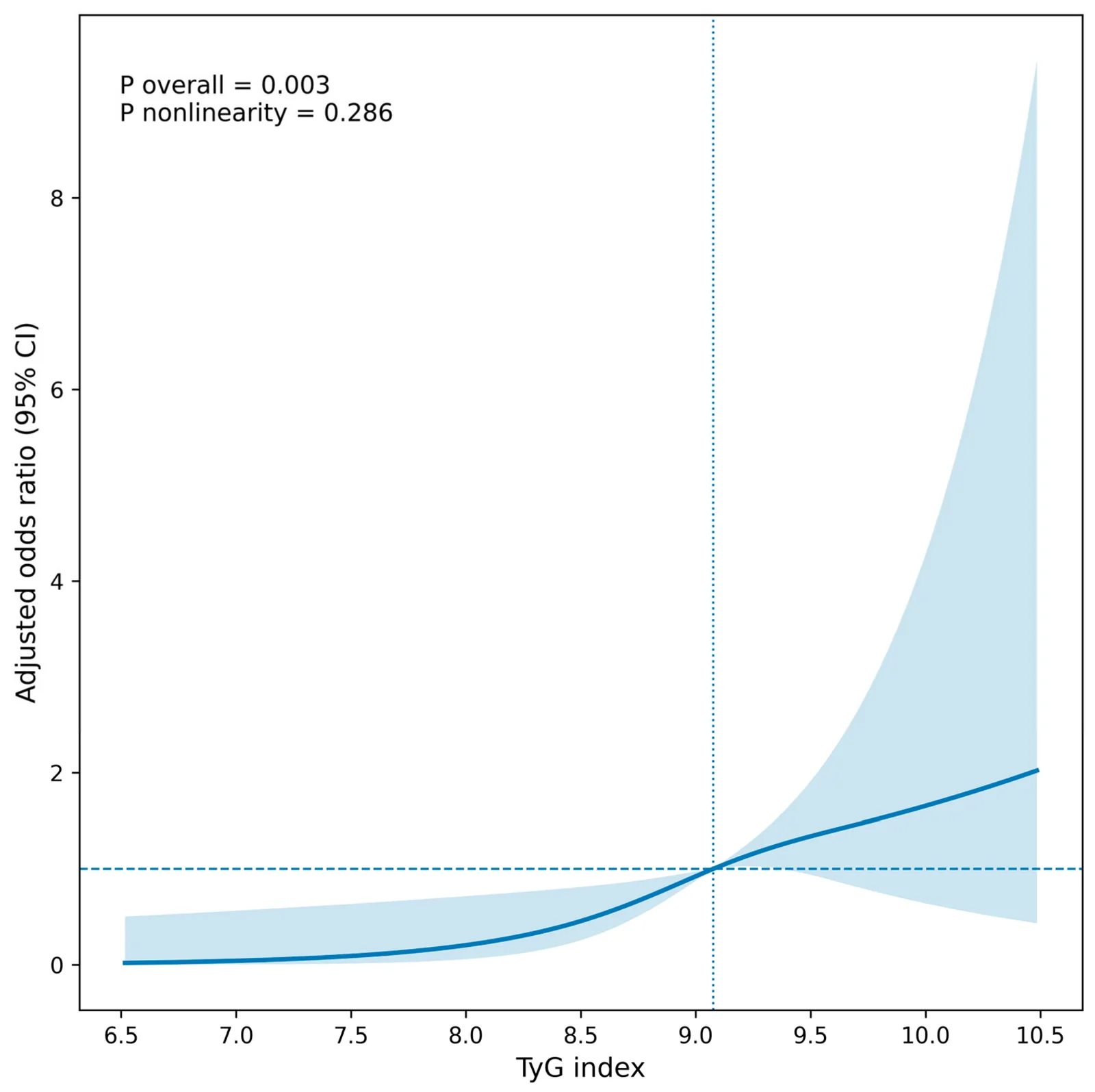
Restricted cubic spline analysis of the association between the TyG index and premature myocardial infarction. The model was adjusted for age, body mass index, smoking status, HDL-C, and serum creatinine and accounted for the matched-pair structure. The association was significant overall (*p* = 0.003), with no evidence of nonlinearity (*p* = 0.286). The solid line represents the adjusted odds ratio, and the shaded area indicates the 95% confidence interval. TyG, triglyceride–glucose; HDL-C, high-density lipoprotein cholesterol. The vertical dashed line represents the median TyG value used as the reference, and the horizontal dashed line represents an odds ratio of 1.0.

Restricted cubic spline analysis demonstrated a significant overall association between the TyG index and premature MI (*p* overall = 0.003). There was no evidence of a significant nonlinear component (*p* for nonlinearity = 0.286), suggesting that the association was compatible with an approximately linear increase in the odds of premature MI across the observed TyG range (Figure 3).

## 4. Discussion

Several important observations emerged from the present study. Higher pre-event TyG levels remained significantly associated with premature MI after propensity score matching and multivariable adjustment. This association was consistent across continuous, tertile-based, and per-standard-deviation analyses, with progressively higher odds of premature MI across increasing TyG categories. Restricted cubic spline analysis demonstrated a significant overall association between the TyG index and premature MI (*p* overall = 0.003), with no evidence of a significant departure from linearity (*p* nonlinearity = 0.286). In contrast, TyG values were not significantly associated with MI subtype, culprit vessel location, or angiographic disease extent. Importantly, the addition of TyG to the clinical model resulted in a modest improvement in discrimination, supported by both DeLong and matched-pair bootstrap analyses.

Premature MI has emerged as an increasingly important cardiovascular health problem in recent years. Although short-term prognosis is generally more favorable in younger patients than in older populations, the long-term risk of recurrent cardiovascular events and mortality remains substantial [18]. Contemporary studies have demonstrated that smoking, dyslipidemia, obesity, and metabolic abnormalities play major roles in the development of premature MI [19,20]. In addition, the increasing prevalence of metabolic risk factors among young adults has been associated with a growing burden of premature CAD [21]. Therefore, evaluation of practical biomarkers reflecting insulin resistance may be particularly relevant in this population.

Earlier investigations have demonstrated that metabolic derangements are common among patients with premature MI and their family members, suggesting that adverse cardiometabolic profiles may contribute substantially to premature CAD [22]. Similarly, insulin resistance-related metabolic abnormalities have been associated with worse cardiovascular outcomes in patients with premature CAD [23]. These observations provide a biological and clinical rationale for investigating insulin resistance surrogates, including the TyG index, in young adults at risk for MI. Shali et al. also found that higher TyG levels were independently associated with early-onset CAD in young adults [24]. Their control group similarly consisted of individuals without significant CAD, although our CCTA-based controls also included mild/non-obstructive plaque.

Previous studies conducted in acute coronary syndrome populations have primarily focused on the prognostic significance of the TyG index and reported associations with adverse cardiovascular outcomes in both NSTEMI and STEMI. In NSTEMI cohorts, higher TyG values have been associated with unfavorable cardiovascular outcomes in non-diabetic patients undergoing PCI and with more advanced CAD [25,26]. Similarly, studies in STEMI populations have linked increased TyG values with vulnerable plaque characteristics, greater atherosclerotic burden, and poorer clinical outcomes [27,28]. In the present premature MI cohort, however, TyG values were comparable across MI subtypes, culprit-vessel locations, and categories of angiographic disease extent. This may partly reflect the distinctive angiographic pattern of premature MI, which is frequently characterized by less diffuse atherosclerosis than that seen in older ACS populations. Consistent with observations by Rallidis et al., single-vessel disease predominated in our cohort, accounting for more than 87% of classifiable cases [2]. The relatively low prevalence of diffuse multivessel disease may therefore have limited the ability to detect differences in TyG according to angiographic severity.

Because diabetes mellitus is closely linked to insulin resistance and directly influences TyG values, we specifically focused on a non-diabetic population. The persistence of the association after PSM and multivariable adjustment is consistent with findings from non-diabetic cohorts, in which higher TyG values have been associated with an unfavorable cardiovascular risk profile and worse outcomes following ACS [29,30,31]. The prognostic relevance of insulin resistance surrogates has also been reported in patients with acute MI [32]. More recently, elevated TyG values have been associated with incident MI in large prospective cohorts and with recurrent MI among young patients with established coronary heart disease [33,34]. Together, these observations suggest that TyG may capture a clinically relevant metabolic phenotype across different stages of coronary disease.

Patients with premature MI and matched controls had virtually identical LDL-C concentrations, whereas HDL-C was modestly lower and triglyceride levels were higher in the premature MI group. These findings suggest that isolated LDL-C measurements may not fully characterize the cardiometabolic risk profile of young adults with premature MI. Previous studies have highlighted the relevance of lipid-related markers beyond LDL-C, including reduced HDL-C and atherogenic lipid patterns, in premature cardiovascular risk [35,36]. In a cohort of non-diabetic ACS patients, Zhang et al. observed poorer cardiovascular outcomes among individuals with elevated TyG levels despite optimal LDL-C control, further supporting the potential complementary information provided by the TyG index [37].

The incremental discrimination analysis provides an additional perspective on the potential clinical relevance of TyG. Addition of the TyG index to the clinical model containing age, BMI, smoking status, HDL-C, and serum creatinine increased the AUC from 0.647 (95% CI 0.598–0.734) to 0.696 (95% CI 0.643–0.775), corresponding to a ΔAUC of 0.050. The DeLong comparison reached statistical significance (*p* = 0.046). Because the cohort was pair-matched, the difference was additionally evaluated using matched-pair bootstrap resampling with 2000 iterations; the 95% CI for ΔAUC excluded zero (0.002–0.096; *p* = 0.034), likewise supporting a modest improvement in discrimination after the addition of TyG. Because several conventional cardiovascular risk factors were used for propensity score matching, these findings should be interpreted as reflecting discrimination within the matched analytical cohort rather than population-level risk prediction. The absolute improvement in discrimination was modest and should not be interpreted as establishing a stand-alone clinical prediction tool. Rather, the consistency of the association across conditional regression, tertile-based, dose–response, spline, and discrimination analyses suggests that TyG may provide complementary metabolic information and merits prospective validation.

### Limitations

This study has several limitations. Its retrospective, single-center design precludes causal inference, while the relatively small matched sample may limit generalizability. Residual confounding may also remain despite propensity score matching and multivariable adjustment. Important determinants of premature MI, particularly family history of premature CAD and lipoprotein(a), were not consistently available and therefore could not be included in the matching or regression models. In addition, insulin resistance was not assessed using established methods such as HOMA-IR or the hyperinsulinemic-euglycemic clamp; therefore, TyG should be regarded as a surrogate marker. The control group consisted of individuals referred for CCTA for clinical indications rather than healthy community-based participants, which may have introduced referral-related selection bias. Moreover, restricting the premature MI group to patients with available pre-event fasting glucose and triglyceride measurements may have introduced healthcare-utilization-related selection bias, as patients with prior laboratory data may differ systematically from those without such data. Finally, although the use of pre-event fasting measurements reduces the influence of the acute MI response, the interval between laboratory assessment and the index MI varied within the prespecified 6-month window, and a single measurement may not fully represent long-term metabolic status.

## 5. Conclusions

Higher pre-event TyG index values were independently associated with premature MI in non-diabetic adults after propensity score matching and multivariable conditional logistic regression. The association showed a graded pattern across TyG levels, with no significant evidence of nonlinearity on restricted cubic spline analysis. The addition of TyG to the clinical model produced a modest but statistically significant improvement in discrimination, supported by both DeLong analysis and matched-pair bootstrap resampling. These findings suggest that the TyG index may serve as a complementary metabolic marker in premature MI; however, its incremental predictive value and clinical utility require confirmation in larger prospective multicenter studies.

## Figures and Tables

**Table 1:** Baseline characteristics of the propensity score-matched non-diabetic cohort.

Variable	Premature MI (*n* = 114)	Control (*n* = 114)	*p* Value
Age, years	43.00 (41.00–46.75)	43.00 (41.00–45.00)	0.490
BMI, kg/m^2^	27.09 (24.38–30.30)	27.36 (25.34–29.62)	0.871
Glucose, mg/dL	116.00 (108.00–125.75)	113.00 (99.25–120.00)	<0.001
Total cholesterol, mg/dL	210.90 (171.20–250.40)	205.50 (185.80–231.30)	0.596
LDL-C, mg/dL	131.96 ± 44.81	131.98 ± 32.36	0.996
HDL-C, mg/dL	41.50 (36.00–46.75)	43.00 (39.00–49.75)	0.034
Triglycerides, mg/dL	165.00 (129.25–199.25)	139.00 (92.50–189.00)	0.011
TyG index	9.18 (8.88–9.40)	8.93 (8.54–9.24)	<0.001
Serum creatinine, mg/dL	0.81 (0.68–1.04)	0.73 (0.65–0.84)	<0.001
Hemoglobin, g/dL	14.40 (13.20–15.47)	14.50 (13.22–15.38)	0.906
Neutrophil count, 10^3^/µL	4.45 (4.01–5.21)	3.83 (3.36–4.87)	0.034
Platelet count, 10^3^/µL	237.50 (202.75–303.75)	244.00 (210.25–282.00)	0.563
Lymphocyte count, 10^3^/µL	2.58 (2.08–3.27)	2.50 (2.04–3.09)	0.267
Monocyte count, 10^3^/µL	0.64 (0.51–0.76)	0.58 (0.47–0.73)	0.011
CRP, mg/dL	0.46 (0.33–0.59)	0.28 (0.20–0.60)	0.240
Male sex, *n* (%)	74 (64.9%)	74 (64.9%)	1.000
Smoking, *n* (%)	76 (66.7%)	78 (68.4%)	0.845
Hypertension, *n* (%)	46 (40.4%)	43 (37.7%)	0.700
Statin use, *n* (%)	21 (18.4%)	25 (21.9%)	0.626

Data are presented as mean ± standard deviation, median (interquartile range), or *n* (%), as appropriate. Comparisons within the matched cohort were performed using the paired-samples *t*-test or Wilcoxon signed-rank test for continuous variables and McNemar’s test for categorical variables, as appropriate. Abbreviations: BMI, body mass index; CRP, C-reactive protein; HDL-C, high-density lipoprotein cholesterol; LDL-C, low-density lipoprotein cholesterol; MI, myocardial infarction; TyG, triglyceride–glucose.

**Table 2:** (**A**) Clinical and angiographic characteristics of patients with premature myocardial infarction. (**B**) Association of the TyG index with myocardial infarction phenotype and angiographic characteristics.

(**A**)			
**Variable**		**Premature MI Patients (*n* = 114)**	
STEMI, *n* (%)		57 (50.0%)	
NSTEMI, *n* (%)		57 (50.0%)	
Culprit artery			
LAD, *n* (%)		56 (49.1%)	
RCA, *n* (%)		28 (24.6%)	
LCX, *n* (%)		25 (21.9%)	
Other, *n* (%)		5 (4.4%)	
Angiographic extent of disease			
Single-vessel disease, *n* (%)		97 (87.4%)	
Multivessel disease (≥2 vessels), *n* (%)		14 (12.6%)	
Revascularization strategy			
PCI, *n* (%)		110 (96.5%)	
CABG, *n* (%)		1 (0.9%)	
Medical therapy, *n* (%)		3 (2.6%)	
(**B**)			
**Variable**	***n***	**TyG Index, Median (IQR)**	***p* Value**
MI phenotype			
STEMI	57	9.09 (8.85–9.37)	
NSTEMI	57	9.23 (8.91–9.46)	0.347
Angiographic disease extent			
Single-vessel disease	97	9.18 (8.87–9.40)	
Multivessel disease (≥2 vessels)	14	9.24 (9.00–9.54)	0.401
Culprit artery			
LAD	56	9.12 (8.87–9.32)	
RCA	28	9.10 (8.93–9.34)	
LCX	25	9.38 (9.00–9.63)	
Other	5	8.91 (8.90–9.27)	0.138

(**A**) Data are presented as *n* (%) unless otherwise specified. Angiographic disease extent was classifiable in 111 patients. Single-vessel disease was defined as involvement of one vessel; multivessel disease was defined as involvement of two or more vessels. Abbreviations: CABG, coronary artery bypass grafting; LAD, left anterior descending artery; LCX, left circumflex artery; MI, myocardial infarction; NSTEMI, non-ST-segment elevation myocardial infarction; PCI, percutaneous coronary intervention; RCA, right coronary artery; STEMI, ST-segment elevation myocardial infarction. (**B**) TyG index values are presented as median (interquartile range). Comparisons were performed using the Mann–Whitney U test or Kruskal–Wallis test, as appropriate. Abbreviations: LAD, left anterior descending artery; LCX, left circumflex artery; MI, myocardial infarction; NSTEMI, non-ST-segment elevation myocardial infarction; RCA, right coronary artery; STEMI, ST-segment elevation myocardial infarction; TyG, triglyceride–glucose.

**Table 3:** Univariable conditional logistic regression analysis for premature myocardial infarction.

Variable	Univariable OR (95% CI)	*p* Value
Age, years	0.998 (0.942–1.058)	0.953
BMI, kg/m^2^	0.993 (0.910–1.083)	0.869
Smoking	0.857 (0.397–1.854)	0.696
Hypertension	1.250 (0.585–2.670)	0.565
Glucose, mg/dL	1.034 (1.013–1.055)	0.001
Total cholesterol, mg/dL	1.001 (0.995–1.007)	0.738
LDL-C, mg/dL	1.000 (0.993–1.007)	0.996
HDL-C, mg/dL	0.971 (0.941–1.002)	0.069
Serum creatinine, mg/dL	16.358 (3.097–86.402)	0.001
Hemoglobin, g/dL	0.982 (0.813–1.185)	0.848
Neutrophil count, 10^3^/µL	1.152 (0.938–1.416)	0.177
Platelet count, 10^3^/µL	1.002 (0.998–1.006)	0.244
Lymphocyte count, 10^3^/µL	1.103 (0.881–1.381)	0.391
Monocyte count, 10^3^/µL	5.233 (1.465–18.688)	0.011
CRP, mg/dL	0.854 (0.390–1.869)	0.692
Statin use	0.810 (0.427–1.534)	0.517
TyG index	2.731 (1.526–4.886)	<0.001

Odds ratios (ORs) and 95% confidence intervals (CIs) were estimated using univariable conditional logistic regression with matched-pair ID as the stratum. Male sex was not estimable because there were no sex-discordant matched pairs. Abbreviations: BMI, body mass index; CI, confidence interval; CRP, C-reactive protein; HDL-C, high-density lipoprotein cholesterol; LDL-C, low-density lipoprotein cholesterol; OR, odds ratio; TyG, triglyceride–glucose.

**Table 4:** Multivariable conditional logistic regression analyses of the TyG index and premature myocardial infarction.

Variable	Model A OR (95% CI)	Model A P	Model B OR (95% CI)	Model B P
TyG index	2.704 (1.408–5.192)	0.003	—	—
T2 vs. T1	—	—	2.859 (1.376–5.940)	0.005
T3 vs. T1	—	—	4.424 (1.859–10.531)	<0.001
Age, years	0.991 (0.926–1.061)	0.803	0.999 (0.931–1.073)	0.984
BMI, kg/m^2^	0.995 (0.901–1.098)	0.918	0.989 (0.888–1.101)	0.835
Smoking	0.616 (0.253–1.500)	0.286	0.608 (0.241–1.537)	0.293
HDL-C, mg/dL	1.010 (0.972–1.050)	0.597	1.009 (0.970–1.050)	0.651
Serum creatinine, mg/dL	14.625 (2.523–84.760)	0.003	13.599 (2.207–83.800)	0.005

Model A included continuous TyG index, age, body mass index, smoking status, HDL-C, and serum creatinine. Model B included TyG tertiles instead of continuous TyG and was adjusted for the same covariates. Conditional logistic regression was performed with matched-pair ID as the stratum. T1 was the reference category. Tertile cut-off values were T1 < 8.84, T2 = 8.84–9.24, and T3 > 9.24. Abbreviations: BMI, body mass index; CI, confidence interval; HDL-C, high-density lipoprotein cholesterol; OR, odds ratio; TyG, triglyceride–glucose.

**Table 5:** Additional analyses of the association between the TyG index and premature myocardial infarction.

Variable	Adjusted OR	95% CI Lower	95% CI Upper	*p* Value
TyG per 1-SD increase	1.696	1.199	2.398	0.003
T1 (lowest tertile)	Reference	—	—	—
T2 vs. T1	2.859	1.376	5.940	0.005
T3 vs. T1	4.424	1.859	10.531	<0.001
P for trend (per tertile increase)	2.159	1.403	3.321	<0.001

Model estimates were obtained using conditional logistic regression with matched-pair ID as the stratum and were adjusted for age, body mass index, smoking status, HDL-C, and serum creatinine. P for trend was calculated by entering TyG tertile categories as an ordinal variable. Tertiles were defined as T1 < 8.84, T2 = 8.84–9.24, and T3 > 9.24. One standard deviation of the TyG index in the matched cohort was 0.531. Abbreviations: CI, confidence interval; OR, odds ratio; SD, standard deviation; TyG, triglyceride–glucose.

**Table 6:** Incremental discrimination analysis of the TyG index for premature myocardial infarction.

Analysis	Estimate	95% CI Lower	95% CI Upper	*p* Value
Clinical model AUC	0.647	0.598	0.734	—
Clinical model + TyG AUC	0.696	0.643	0.775	—
ΔAUC, DeLong analysis	0.050	—	—	0.046
ΔAUC, matched-pair bootstrap	0.050	0.002	0.096	0.034

The clinical model included age, body mass index, smoking status, HDL-C, and serum creatinine. Incremental discrimination after the addition of the TyG index was assessed using DeLong’s test. Because the study used a matched case-control design, ΔAUC was additionally evaluated using matched-pair bootstrap resampling with 2000 iterations. Both the DeLong comparison and matched-pair bootstrap analysis supported a modest incremental improvement in discrimination. Abbreviations: AUC, area under the receiver operating characteristic curve; CI, confidence interval; TyG, triglyceride–glucose.

## Data Availability

The data presented in this study are available on request from the corresponding author due to institutional policies and patient confidentiality considerations.

## Supplementary Materials

The following supporting information can be downloaded at: https://www.mdpi.com/article/10.3390/diagnostics16182999/s1, Table S1: Standardized mean differences before and after propensity score matching.
