# Supplementary material for: Association of the Triglyceride–Glucose Index with Premature Myocardial Infarction in a Propensity Score-Matched Non-Diabetic Population
Source: Diagnostics (Basel). 2026 Sep 16;16(18):2999. doi: 10.3390/diagnostics16182999 (PMC13605665; doi:10.3390/diagnostics16182999)
Supplement: Supplementary file 1 [file diagnostics-16-02999-s001.zip › diagnostics-4535205-supplementary.pdf]

Supplementary Table S1. Standardized mean differences before and after propensity score matching.

Variable	Before matching SMD	After matching SMD
Age, years	0.164	0.006
BMI, kg/m²	0.196	0.016
Glucose, mg/dL	0.538	0.490
Total cholesterol, mg/dL	0.105	0.043
LDL-C, mg/dL	0.098	0.001
HDL-C, mg/dL	0.550	0.228
Triglycerides, mg/dL	0.399	0.335
TyG index	0.624	0.499
Serum creatinine, mg/dL	0.512	0.506
Hemoglobin, g/dL	0.259	0.020
Neutrophil count, 10^3/µL	0.140	0.148
Platelet count, 10^3/µL	0.222	0.160
Lymphocyte count, 10^3/µL	0.041	0.119
Monocyte count, 10^3/µL	0.002	0.349
CRP, mg/dL	0.217	0.024
Male sex	0.480	0.000
Smoking	0.458	0.037
Hypertension	0.402	0.054
Statin use	0.003	0.088

Abbreviations: BMI, body mass index; CRP, C-reactive protein; HDL-C, high-density lipoprotein cholesterol; LDL-C, low-density lipoprotein cholesterol; SMD, standardized mean difference; TyG, triglyceride-glucose index.

Note: Age, sex, BMI, smoking, and hypertension were included in the propensity score model. An absolute SMD <0.10 was considered indicative of adequate balance for covariates included in the matching procedure. Other variables are shown descriptively and were not matching covariates.
